# HemU and TonB1 contribute to hemin acquisition in *Stenotrophomonas maltophilia*

**DOI:** 10.3389/fcimb.2024.1380976

**Published:** 2024-03-26

**Authors:** Chun-Hsing Liao, Hsu-Feng Lu, Ching-Wei Yang, Ting-Yu Yeh, Yi-Tsung Lin, Tsuey-Ching Yang

**Affiliations:** ^1^ Division of Infectious Disease, Far Eastern Memorial Hospital, New Taipei City, Taiwan; ^2^ Department of Medicine, National Yang Ming Chiao Tung University, Taipei, Taiwan; ^3^ Department of Medical Laboratory Science and Biotechnology, Asia University, Taichung, Taiwan; ^4^ Department of Biotechnology and Laboratory Science in Medicine, National Yang Ming Chiao Tung University, Taipei, Taiwan; ^5^ Division of Infectious Diseases, Department of Medicine, Taipei Veterans General Hospital, Taipei, Taiwan; ^6^ School of Medicine, National Yang Chiao Tung University, Taipei, Taiwan

**Keywords:** *Stenotrophomonas maltophilia*, hemin acquisition, ExbB-ExbD-TonB complex, HemU, Fur, HemP

## Abstract

**Introduction:**

The hemin acquisition system is composed of an outer membrane TonB-dependent transporter that internalizes hemin into the periplasm, periplasmic hemin-binding proteins to shuttle hemin, an inner membrane transporter that transports hemin into the cytoplasm, and cytoplasmic heme oxygenase to release iron. Fur and HemP are two known regulators involved in the regulation of hemin acquisition. The hemin acquisition system of *Stenotrophomonas maltophilia* is poorly understood, with the exception of HemA as a TonB-dependent transporter for hemin uptake.

**Methods:**

Putative candidates responsible for hemin acquisition were selected via a homolog search and a whole-genome survey of *S. maltophilia*. Operon verification was performed by reverse transcription-polymerase chain reaction. The involvement of candidate genes in hemin acquisition was assessed using an in-frame deletion mutant construct and iron utilization assays. The transcript levels of candidate genes were determined using quantitative polymerase chain reaction.

**Results:**

*Smlt3896-hemU-exbB2-exbD2-tonB2* and *tonB1-exbB1-exbD1a-exbD1b* operons were selected as candidates for hemin acquisition. Compared with the parental strain, *hemU* and *tonB1* mutants displayed a defect in their ability to use hemin as the sole iron source for growth. However, hemin utilization by the *Smlt3896* and *tonB2* mutants was comparable to that of the parental strain. *HemA* expression was repressed by Fur in iron-replete conditions and derepressed in iron-depleted conditions. HemP negatively regulated *hemA* expression. Like *hemA*, *hemU* was repressed by Fur in iron-replete conditions; however, *hemU* was moderately derepressed in response to iron-depleted stress and fully derepressed when hemin was present. Unlike *hemA* and *hemU*, the *TonB1-exbB1-exbD1a-exbD1b* operon was constitutively expressed, regardless of the iron level or the presence of hemin, and Fur and HemP had no influence on its expression.

**Conclusion:**

HemA, HemU, and TonB1 contribute to hemin acquisition in *S. maltophilia*. Fur represses the expression of *hemA* and *hemU* in iron-replete conditions. *HemA* expression is regulated by low iron levels, and HemP acts as a negative regulator of this regulatory circuit. *HemU* expression is regulated by low iron and hemin levels in a *hemP*-dependent manner.

## 1 Introduction

Iron is an essential element for almost all bacteria because of its role as a cofactor for metabolic enzymes and components of the electron transport chain (Jordan and Reichard, 1998; Imlay, 2008; Roux et al., 2009). Owing to its low solubility, most ferric iron in mammalian hosts is bound by heme groups and other iron-binding proteins (Stojiljkovic and Perkins-Balding, 2002). Hemin is the most abundant source of iron for bacterial pathogens in their hosts (Choby and Skaar, 2016). To survive in infectious niches, pathogens have evolved several hemin acquisition systems that ensure iron availability. The hemin acquisition system is generally equipped with outer membrane TonB-dependent transporters (TBDTs) to internalize extracellular hemin into the periplasm, periplasmic proteins to shuttle hemin, inner membrane ABC transporters to transport hemin from the periplasm into the cytosol, and cytoplasmic proteins to provide ATP and degrade hemin (Wandersman and Delepelaire, 2004; Runyen-Janecky, 2013). Hemin uptake via TBDT is energy-dependent, and energy is transduced from the inner to the outer membrane using the TonB/ExbB/ExbD system (Krewulak and Vogel, 2011). Known hemin acquisition systems include *hemPRST* of *Yersinia enterocolitica*, *hmuRSTUV* of *Yersinia pestis*, *phuRSTUV* of *Pseudomonas aeruginosa*, *shmR-hmuPSTUV* of *Ensifer meliloti*, *hmuR-hemP-hmuTUV* of *Bradyrhizobium japonicum*, *hemP-hmuRSTUV* of *Burkholderia multivorans*, and *hemP-hemA* of *S. maltophilia*. A comparison of these hemin acquisition systems was summarized in a recent study (Shih et al., 2022). Generally, genes encoding TBDTs, periplasmic hemin transporter proteins, inner membrane permease proteins, and ATP-binding proteins are clustered or organized into an operon.

In most bacteria, ferric uptake regulator (Fur) is the master regulator of iron hemostasis (Troxell and Hassan, 2013). As a repressor, the Fur-Fe^2+^ complex binds to the consensus DNA sequence, designated as a Fur box, to exclude RNA polymerase binding, which blocks transcriptional initiation. When the intracellular ferrous iron level is too low to bind with Fur, Fur dissociates from the Fur box, triggering the expression of Fur-regulated genes. In addition to Fur, HemP/HmuP is another regulator known to be involved in hemin acquisition. The transcription of *hemP/hmuP* is repressed by Fur under iron-replete conditions. In iron-depleted conditions, HemP/HmuP is upregulated and functions as a transcription factor contributing to TBDT expression in alpha- and beta-proteobacteria (Sato et al., 2017).

*Stenotrophomonas maltophilia* is an opportunistic environmental bacterium that can cause broad-spectrum infections of the respiratory tract, soft tissue, urinary tract, eyes, and wounds. *S. maltophilia* infections are difficult to treat because the pathogen exhibits intrinsic and acquired resistance to a broad spectrum of antibiotics (Liu et al., 2023). To adapt to diverse environments, *S. maltophilia* is equipped with several iron acquisition systems for the utilization of ferric iron, ferrous iron, hemin, ferric citrate, and *Pseudomonas aeruginosa* pyochelin (Nas and Cianciotto, 2017; Kalidasan et al., 2018; Liao et al., 2022; Pan et al., 2022; Shih et al., 2022). Hemin is the most abundant source of iron for pathogens in the infection niche; hence, hemin acquisition is a critical determinant of pathogen virulence. The involvement of *hemP-hemA-smlt0796-smlt0797* and *Smlt2357-Smlt2356-Smlt2355* clusters (the homologs of *hmuV*, *hmuU*, and *hmuT*, respectively) in hemin acquisition was evaluated in a recent study (Shih et al., 2022). Of the seven genes, *hemP* and *hemA* are involved in hemin acquisition. *smlt0796*, *smlt0797*, *smlt2357, smlt2356*, and *smlt2355* are not individually critical for hemin acquisition. HemA is a TBDT for hemin uptake. Distinct from the known HemP/HmuP in alpha- and beta-proteobacteria, HemP of *S. maltophilia* negatively regulates *hemA* expression in iron-depleted conditions (Shih et al., 2022). In this study, we aimed to further elucidate the other members, in addition to *hemA* and *hemP*, responsible for hemin acquisition in *S. maltophilia*.

## 2 Materials and methods

### 2.1 Bacterial strains and plasmids

The bacterial strains and plasmids used in this study are listed in **Supplementary Table S1**.

### 2.2 Construction of in-frame deletion mutants

The double-crossover homologous recombination strategy was used to construct in-frame deletion mutants, as described previously (Yang et al., 2009). Briefly, two DNA fragments, upstream and downstream of the gene intended for deletion, were amplified from the chromosome of *S. maltophilia* KJ via polymerase chain reaction (PCR). The primer sets are listed in **Supplementary Table S2**. The two PCR amplicons were cloned into the pEX18Tc vector to generate the recombinant plasmids listed in **Supplementary Table S1**. The pEX18Tc-derived plasmids were introduced into *S. maltophilia* KJ or KJΔEnt via conjugation. Transconjugants were selected on LB plates containing 1.5 μg/mL norfloxacin and 30 μg/mL tetracycline. Then, the selected transconjugants were transferred to LB containing 10% sucrose for the selection of deletion mutant. The correctness of deletion mutants was checked by PCR and sequencing. Double mutant and triple mutant were constructed sequentially form the single mutant by the same procedure.

### 2.3 Reverse transcription-PCR and operon verification

**Figure 1:**
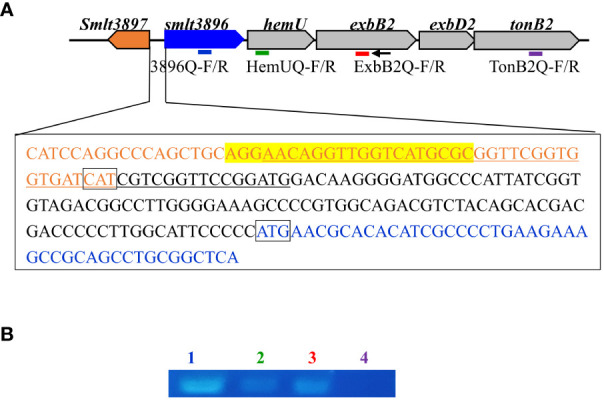
Genetic organization and operon verification of the *smlt3896-hemU-exbB2-exbD2-tonB2* cluster of *Stenotrophomonas maltophilia*. **(A)** Genetic organization of the *hemO-hemU-exbB2-exbD2-tonB2* cluster of *S. maltophilia*. The orientation of the genes is indicated by arrows. The small black arrow indicates the locations of the ExbB2-C primer used for reverse transcription. The bars indicate the polymerase chain reaction (PCR) amplicons obtained using primer sets 3896Q-F/R (blue), HemUQ-F/R (green), ExbB2Q-F/R (red), and TonB2Q-F/R (purple). The DNA sequences encoding the N-termini of *smlt3897* and *smlt3896*, and the intergenic region of *smlt3897* and *smlt3896* are placed in a rectangle. *smlt3897* and *smlt3896* genes are marked in orange and blue, respectively. The putative promoter of the *smlt3896-hemU-exbB2-exbD2-tonB2* operon is underlined according to the prediction made using the tool at https://www.fruitfly.org/seq_tools/promoter.html. The putative Fur box is marked in yellow, based on the previously reported Fur box sequence. **(B)** Operon analysis of the *smlt3896-*hemU*-exbB2-exbD2-tonB2* cluster. DNA-free RNA was prepared from logarithmical phase *S. maltophilia* KJ cells treated with 50 mg/mL 2, 2’-dipyridyl and 150 μM hemin. cDNAs were obtained by reverse transcription using the primer ExbB2-C and then used as the template for PCR. PCR amplicons were separated by agarose gel electrophoresis and stained with ethidium bromide. Lane 1, PCR amplicon generated by 3896Q-F and 3896Q-R; Lane 2, PCR amplicon generated by HemUQ-F and HemUQ-R; Lane 3, PCR amplicon generated by ExbB2Q-F and ExbB2Q-R; Lane 4, PCR amplicon generated by TonB2Q-F and TonB2Q-R (negative control).

An overnight culture of KJ cells was inoculated into fresh LB containing 50 μg/mL 2, 2’-dipyridyl (DIP) and 150 μM hemin at an initial OD_450_ of 0.15. After a 15-h incubation at 37°C, DNA-free RNA was prepared and reverse transcribed into cDNA using the primer ExbB2-C (**Supplementary Table S2**, **Figure 1A**). cDNA was used as the template for PCR using the primer sets 3896Q-F/R, HemUQ-F/R, ExbB2Q-F/R, and TonB2Q-F/R (**Supplementary Table S2**, **Figure 1A**). TonB2Q-F/R primer sets were used as a control for DNA contamination check.

### 2.4 Iron utilization assay

An overnight culture of bacteria was inoculated into fresh LB at an initial OD_450_ of 0.15. After a 5-h incubation at 37°C, logarithmic-phase bacterial cells were adjusted to 2 × 10^5^ CFU/μL and then serially 10-fold diluted. A 5-µL bacterial aliquot was spotted onto Luria-Bertani (LB) agar containing different compounds as indicated. Cell viability was observed after 24 h of incubation at 37°C. The experiment was performed in triplicate.

### 2.5 Construction of complementation plasmids pHemU and pTonB1

The *hemU* and *tonB1* genes were amplified from *S. maltophilia* KJ using the primer sets HemU-F/R and TonB1-F/R (**Supplementary Table S1**), and cloned into pRK415 to yield pHemU and pTonB1, respectively.

### 2.6 Quantitative real-time PCR

An overnight culture of KJ, KJΔFur, and KJΔHemP was inoculated into fresh LB with or without additives as indicated at an initial OD_450_ of 0.15. After a 5-h incubation at 37°C, DNA-free RNA was prepared and reverse transcribed to cDNA using random hexamers primers. *hemA*, *hemU*, and *tonB1* transcript levels were then determined. The primers used are listed in **Supplementary Table S2**. *16S rRNA* was used as an internal control for the normalization of gene expression levels. Fold change was calculated using the *ΔΔC_T_* method (Livak and Schmittgen, 2001).

### 2.7 Bioinformatics assay

A protein homolog search was carried out using the BLASTp tool from the NCBI (https://blast.ncbi.nlm.nih.gov/Blast.cgi) against *S. maltophilia* K279a genome (Accession no. AM74169.1). Protein sequences comparison was performed using the Global Align tool from the NCBI (https://blast.ncbi.nlm.nih.gov/Blast.cgi). The putative promoter was predicted using the website https://www.fruitfly.org/seq_tools/promoter.html.

### 2.8 Statistical analysis

Student’s *t* test was used for comparison of means between the groups. The Bonferroni correction method was applied to adjust the *P* values.

## 3 Results

### 3.1 *smlt3896*, *hemU*, *exbB2*, *exbD2*, and *tonB2* form an operon

To elucidate the other candidates responsible for hemin acquisition, *in silico* analysis of the *S. maltophilia* K279a genome (Crossman et al., 2008) revealed a five-gene cluster, *Smlt3896-Smlt3892*, which, when combined, was predicted to encode a system for hemin acquisition (**Figure 1A**). *Smlt3896* encodes a 195-aa cytosolic protein that is homologous to *Pseudomonas aeruginosa* PigA (Ratliff et al., 2001) (53% similarity, 41% identity), *Pseudomonas aeruginosa* BphO (Wegele et al., 2004) (40% similarity, 31% identity), *Neisseria meningitidis* HemO (Zhu et al., 2000) (49% similarity, 34% identity), and *Corynebacterium diphtheriae* HemO (Wilks and Schmitt, 1998) (39% similarity, 23% identity). PigA, BphO, and HemO are heme oxygenases that catalyze the degradation of hemin to produce biliverdin, ferrous iron, and carbon monoxide. The protein encoded by *Smlt3895* was predicted to be a 155-aa inner membrane transmembrane protein and displayed no significant similarity with the proteins of known functions. The proteins encoded by *Smlt3894*, *Smlt3893*, and *Smlt3892* were annotated as ExbB, ExbD, and TonB, respectively. The ExbB-ExbD-TonB complex is an energy transducer embedded in the inner membrane that provides ATP to support the function of TonB-dependent outer membrane proteins (Koebnik, 2005). Recently, a *tonB* gene (*Smlt0009*) was identified in *S. maltophilia* and characterized by Calvopiña et al (Calvopiña et al., 2020). Furthermore, the inner membrane permeases for known hemin acquisition systems in other bacteria are annotated as HemU, HmuU, or PhuU (Shih et al., 2022). Based on the following results and to avoid confusion with *Smlt0009*, we designated the five genes *smlt3896*, *hemU*, *exbB2*, *exbD2*, and *tonB2*. A short intergenic region (31 bp) between *smlt3896* and *hemU*, and a 29-bp overlap between *hemU* and *exbB2* were noticed (**Figure 1A**). A promoter prediction tool (https://www.fruitfly.org/seq_tools/promoter) demonstrated that a putative promoter located upstream of *smlt3896*, but not upstream of *hemU* and *exbB2*. Furthermore, *exbB*, *exbD*, and *tonB* are generally organized as an operon (Ratliff et al., 2022). Thus, we speculated that *smlt3896*, *hemU*, *exbB2*, *exbD2*, and *tonB2* form an operon. We used revers transcription (RT) to investigate whether *smlt3896*, *hemU*, and *exbB2* were co-transcribed. Considering that hemin acquisition-associated genes are generally expressed under iron-depleted conditions, we prepared DNA-free RNA from the DIP-treated KJ cells, and RT was carried out using the primer ExbB2-C. No positive results were obtained; therefore, we sought to determine whether hemin is a crucial inducer of expression of the five-gene cluster. DNA-free RNA prepared from KJ cells treated with 50 μg/mL DIP and 150 μM hemin was used as a template for operon verification. The results demonstrated that *smlt3896*, *hemU*, and *exbB2* locate in a single transcript (**Figure 1B**) and they can be part of the *smlt3896-hemU-exbB2-exbD2-tonB2* operon.

### 3.2 HemU contributes to hemin utilization

**Figure 2:**
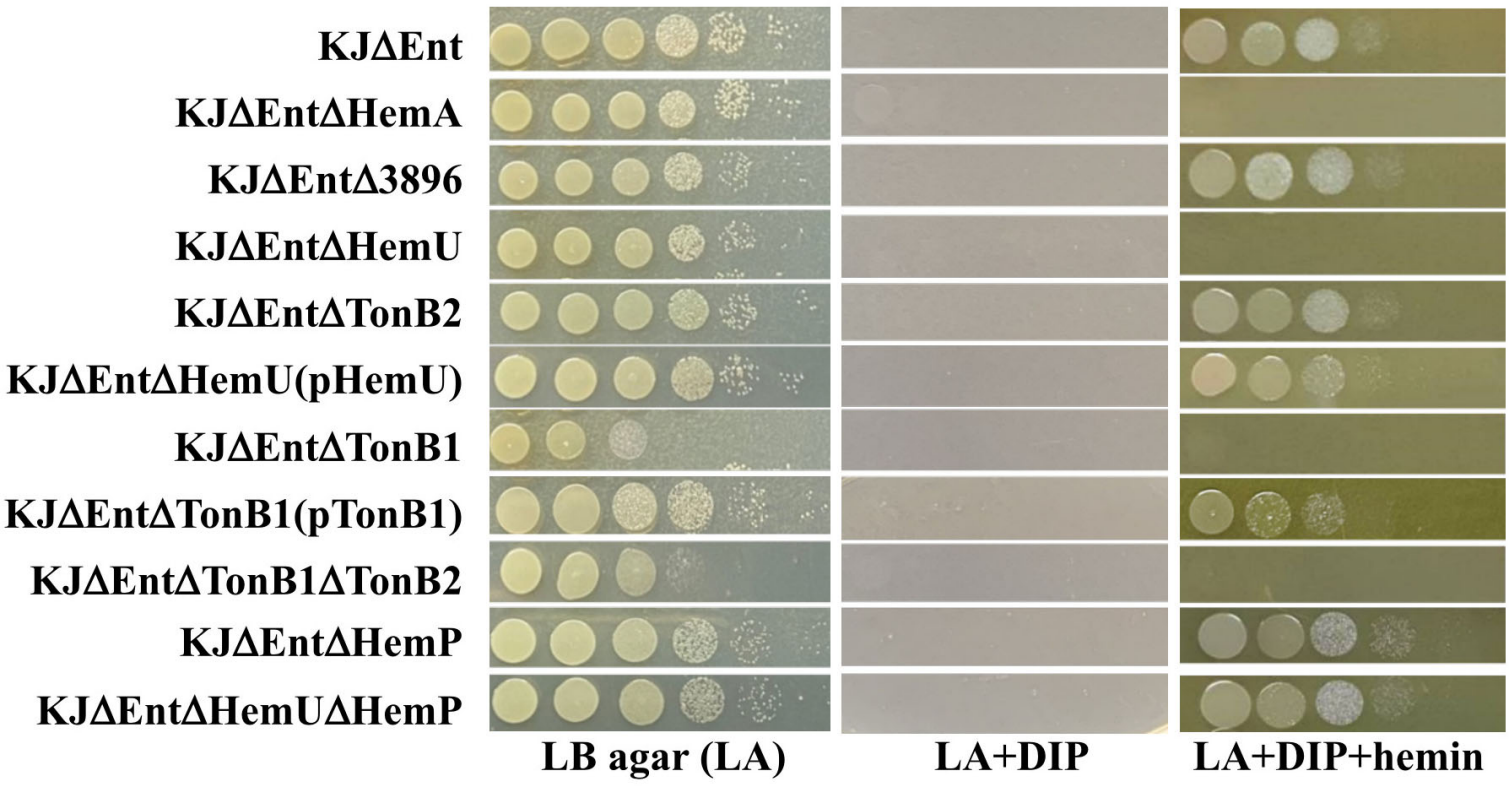
Cell viability of parental strain KJΔEnt, its derived mutants, and complementation strains under iron-replete (LA), iron-depleted (LA + DIP), and iron-depleted with hemin as sole iron source (LA + DIP + hemin). Bacterial cells (2 × 10^5^ CFU/μL) were 10-fold serially diluted and a 5-μL aliquot was spotted onto Luria-Bertani (LB) agar as indicated. After 24 h of incubation at 37°C, the bacteria were imaged to assess their viability. The experiment was performed in triplicate, and one was selected as a representative. 2, 2’-dipyridyl (DIP), 50 μg/mL; hemin, 150 μM.

To eliminate the growth bias caused by stenobactin-mediated iron acquisition in iron-depleted media, KJΔEnt, a stenobactin-null mutant (Pan et al., 2022), was chosen as the parental strain for the following experiments. An array of in-frame deletion mutants was constructed using KJΔEnt to generate KJΔEntΔ3896, KJΔEntΔHemU, and KJΔEntΔTonB2. The resulting mutants and their parental strain, KJΔEnt, were subjected to an iron utilization assay, using hemin as the sole iron source. Of the three mutants tested, only KJΔEntΔHemU lost viability in DIP- and hemin-containing medium (**Figure 2**, **Supplementary Figure S1A**), even though the incubation time was prolonged to 48 h. To assess whether *hemU* deletion causes a polar effect on the expression of downstream genes, the *tonB2* transcript levels in KJΔEnt and KJΔEntΔHemU were determined by qRT-PCR. The results demonstrated that KJΔEnt and KJΔEntΔHemU exhibited comparable *tonB2* transcript levels. Furthermore, a *hemU* complementation strain KJΔEntΔHemU(pHemU) restored the ability to use hemin as an iron source for growth (**Figure 2**, **Supplementary Figure S1B**). Collectively, HemU contributes to hemin utilization.

In addition to hemin, we considered the involvement of the *smlt3896-*hemU*-exbB2-exbD2-tonB2* operon in ferric citrate and ferristenobactin utilization. These results tentatively ruled out the contribution of the *smlt3896-hemU-exbB2-exbD2-tonB2* operon to ferric citrate and ferri-stenobactin utilization (**Supplementary Figure S2**).

### 3.3 TonB1 (Smlt0009) contributes to hemin utilization

A more interesting finding from the above results (**Figure 2**) was that *exbB2*, *exbD2*, and *tonB2* do not contribute to hemin acquisition, although they and *hemU* are in the same operon (**Figure 1**). Considering this functional redundancy, we tested whether other TonB systems were involved in hemin acquisition. Another *tonB*-associated cluster, Smlt0009-Smlt0012, was reported by Calvopina et al (Calvopiña et al., 2020). One peculiar observation was the presence of two *exbD* genes in the four-gene cluster. We annotated the four genes as *tonB1*, *exbB1*, *exbD1a*, and *exbD1b* (**Supplementary Figure S3**). The protein sequence identities and similarities between the two *exbB-exbD-tonB* clusters are summarized in **Supplementary Figure S3**. To assess the role of TonB1 in hemin acquisition, *tonB1* deletion mutant and *tonB1/tonB2* double mutants were generated in KJΔEnt to yield KJΔEntΔTonB1 and KJΔEntΔTonB1ΔTonB2, respectively. KJΔEntΔTonB1 displayed compromised growth on LB agar, which is consistent with previous observations (Calvopiña et al., 2020). Furthermore, KJΔEntΔTonB1 and KJΔEntΔTonB1ΔTonB2 showed no visible viability in DIP- and hemin-containing media (**Figure 2**, **Supplementary Figure S1C**). Complementation of KJΔTonB1 with an intact *tonB1* gene partially restored the ability to utilize hemin for growth in iron-depleted conditions (**Figure 2**, **Supplementary Figure S1C**). Collectively, TonB1 contributes to hemin utilization.

Based on the above results, *tonB1*, but not *tonB2*, contributed to hemin utilization (**Figure 2**). We wondered whether the deletion of *tonB2* was complemented by an increase in *tonB1* expression. To test this notion, the *tonB1* transcript levels in KJΔEnt and KJΔEntΔTonB2 were determined by qRT-PCR. A comparable *tonB1* transcript level was observed in KJΔEnt and KJΔEntΔTonB2.

### 3.4 Regulation of smlt3896-hemU-exbB2-exbD2-tonB2 and tonB1-exbB1-exbD1a-exbD1b operons

**Figure 3:**
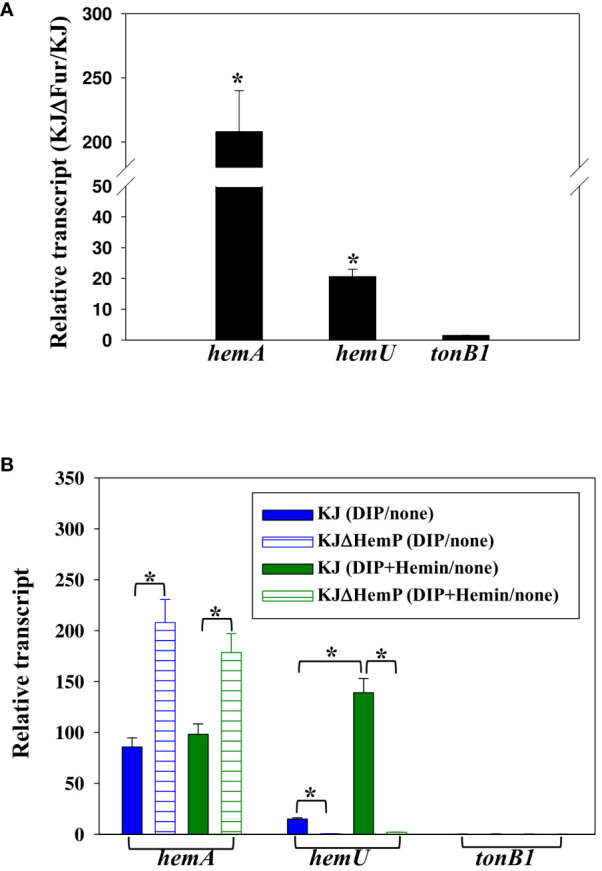
Regulation of *hemA, hemU*, and t*onB1* expression. Data are the means from three independent experiments. The error bars represent the standard deviations for triplicate samples. *, *P* ≤ 0.05 (significance calculated by Student’s t test). **(A)** Role of Fur in the expression of *hemA*, *hemU*, and *tonB1*. KJ and KJΔFur were cultured overnight and inoculated into fresh LB medium at an initial OD_450_ of 0.15. After a 15-h incubation, the *hemA*, *hemU*, and *tonB1* transcript levels were determined by quantitative RT-PCR. The relative transcript levels were calculated based on the transcript level of KJ cells assigned as 1. **(B)** Impact of HemP, iron level, and hemin on the expression of *hemA*, *hemU*, and *tonB1*. KJ and KJΔHemP cells were cultured overnight and inoculated into fresh LB medium with or without additives as indicated. After 15 h of incubation, *hemA*, *hemU*, and *tonB1* transcript levels were determined by quantitative RT-PCR. For DIP treatment, the DIP concentration was 30 μg/mL. For DIP and hemin treatment, the concentrations of DIP and hemin added were 50 μg/mL and 150 μM, respectively. The relative transcript levels were calculated based on the transcript level in the counterpart without additives assigned as 1.

The contributions of *hemA* and *hemP* to hemin acquisition have previously been reported (Shih et al., 2022). The above results support the involvement of *hemU* and *tonB1* in hemin acquisition (**Figure 2**). Fur and HemP are two known regulators participating in hemin acquisition; thus, we were interested in investigating the regulatory roles of Fur and HemP in the expression of *tonB1-exbB1-exbD1a-exbD1b* and *smlt3896-hemU-exbB2-exbD2-tonB2* operons. For convenience, the expression of the *hemA-hemP-Smlt0796-Smlt0797* operon, described in our previous study (Shih et al., 2022), is also shown in **Figure 3** for comparison.

First, we sought to determine whether a putative Fur box is present upstream of the three operons, and positive results were found in the *hemA-hemP-Smlt0796-Smlt0797* and *smlt3896-hemU-exbB2-exbD2-tonB2* operons (Shih et al., 2022). To further confirm the regulatory role of Fur in the expression of the three operons, the *hemA*, *hemU*, and *tonB1* transcript levels of KJ and KJΔFur, a *fur* deletion mutant (Liao et al., 2020), were determined by quantitative RT-PCR. As expected, the levels of *hemA* and *hemU* transcripts, but not the *tonB1* transcript, were increased in KJΔFur (**Figure 3A**), verifying the repressor role of Fur in the expression of the *hemA-hemP-Smlt0796-Smlt0797* and *smlt3896-*hemU-*exbB2-exbD2-tonB2* operons.

In a recent study, we verified that the transcription factor HemP negatively regulates the expression of *hemA* (Shih et al., 2022). To investigate whether the three operons were regulated by HemP, iron levels, and hemin, the *hemA*, *hemU*, and *tonB1* transcripts of KJ and KJΔHemP, with or without DIP and hemin, were determined by quantitative RT-PCR. The *hemA* transcript of wild-type KJ showed an approximately 90-fold increase in its DIP- and DIP/hemin-additive counterparts (**Figure 3B**). Furthermore, the upregulation level of the *hemA* transcript was more significant in the KJΔHemP group (**Figure 3B**). These observations indicated that the *hemA-hemP-Smlt0796-Smlt0797* operon was repressed by Fur in iron-replete conditions and derepressed in response to low iron levels, regardless of the presence of hemin. HemP functions as a negative transcription factor for the expression of the *hemA-hemP-Smlt0796-Smlt0797* operon.

Unlike *hemA* expression, *hemU* transcript levels in wild-type KJ showed an approximately 15-fold increase upon DIP challenge and a 139-fold increase in the presence of DIP and hemin (**Figure 3B**), indicating that hemin is a crucial determinant of the full induction of *the smlt3896-*hemU*-exbB2-exbD2-tonB2* operon. This observation provides a reasonable explanation for the detection of the *smlt3896-hemU-exbB2-exbD2-tonB2* transcript in DIP- and hemin-treated KJ cells, but not in DIP-treated KJ cells (**Figure 1B**). In addition, we observed that the DIP- and hemin-mediated upregulation of *hemU* was not observed in KJΔHemP cells (**Figure 3B**), indicating that the expression of the *smlt3896-hemU-exbB2-exbD2-tonB2* is dependent on HemP.

A similar strategy was used to study the expression of the *tonB1-exbB1-exbD1a-exbD1b* operon. Of note, the *tonB1-exbB1-exbD1a-exbD1b* operon was intrinsically highly expressed and its expression level was not affected by Fur, HemP, iron levels, or hemin.

An interesting observation attracted our attention. Based on the findings in this study, HemU contributed to hemin utilization (**Figure 2**) and its expression was HemP-dependent (**Figure 3B**). Expectedly, a *hemP* mutant should be defective in hemin utilization in iron-depleted conditions. However, in our previous study, we showed that *hemP* deletion has no impact on hemin utilization (Shih et al., 2022, **Figure 2**, **Supplementary Figure S1D**). To elucidate the contradiction, we constructed a *hemU* and *hemP* mutant in a stenobactin-null background, KJΔEntΔHemUΔHemP, for hemin utilization evaluation. Interestingly, KJΔEntΔHemU lost the ability of hemin utilization in iron-depleted condition, but KJΔEntΔHemUΔHemP reverted this ability (**Figure 2**, **Supplementary Figure S1D**), indicating that a *hemU*-independent mechanism responsible for hemin utilization in *hemP* mutant.

## 4 Discussion

**Figure 4:**
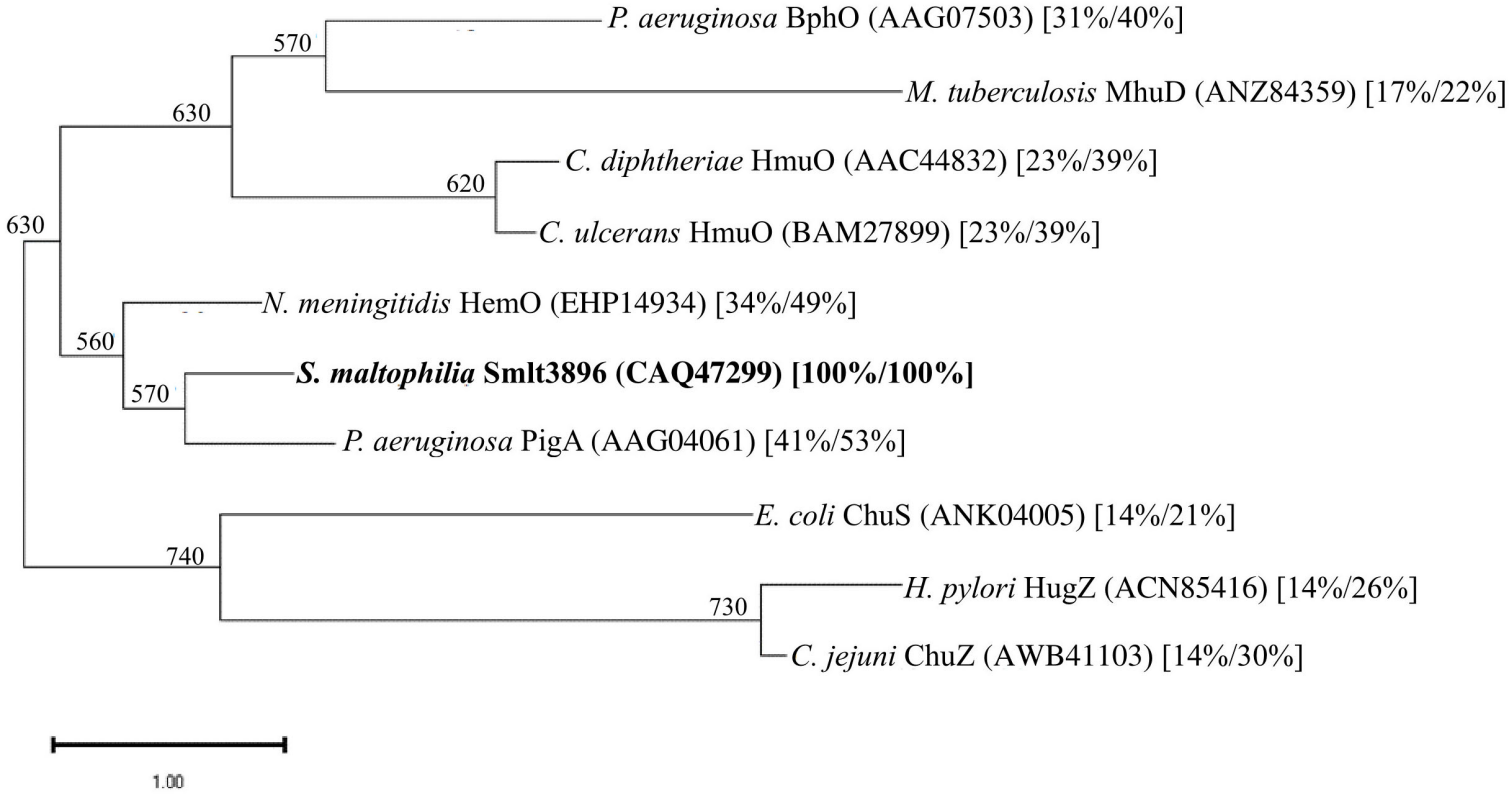
Phylogenetic analysis of *S. maltophilia* Smlt3896 and known HemO proteins of other bacteria. A dendrogram was constructed with the amino acid sequences of the proteins using the neighbor-joining method. The numbers below the branches indicate the number of bootstraps, which were calculated from 1,000 replicates. The protein accession numbers are included in brackets. The numbers in square brackets are the protein identity and similarity values compared with Smlt3896.

Hemin is an important source of iron in infection niches, and it is utilized by most pathogens. Extracellular hemin can be directly captured by pathogens and transported into the cytoplasm via the outer membrane TBDT, periplasmic transport proteins, and inner membrane transporters. Once hemin is located in the cytoplasm, it must be released for further utilization by the pathogen. Heme oxygenase (HemO) is an enzyme involved in this process. Heme oxygenase catalyzes the NADPH-reductase-dependent cleavage of hemin into biliverdin, carbon monoxide, and free iron (Frankenberg-Dinkel, 2004; Wilks and Burkhard, 2007). Heme oxygenases have been identified in several pathogenic bacteria, including *Corynebacterium* spp. HmuO (Schmitt, 1997; Wilks and Schmitt, 1998), *Neisseria meningitidis* HemO (Zhu et al., 2000), *P. aeruginosa* PigA and BphO (Ratliff et al., 2001; Wegele et al., 2004), *E. coli* ChuS (Suits et al., 2005), *Helicobacter pylori* HugZ (Guo et al., 2008), *Campylobacter jejuni* ChuZ (Zhang et al., 2011), and *Mycobacterium tuberculosis* MhuD (Nambu et al., 2013). However, the protein sequence identities among these heme oxygenases are not high. Phylogenetic analysis of Smlt3896 and the known HemO proteins of other bacteria revealed that Smlt3896 phylogenetically clustered with *N. meningitidis* HemO and *P. aeruginosa* PigA (**Figure 4**). We found that KJΔEntΔ3896 was able to grow using hemin as an iron source (**Figure 2**), even though Smlt3896 was annotated as *hemO* in the sequenced K279a genome and was phylogenetically close to known HemO proteins (**Figure 4**). This observation led us to speculate that heme oxygenase redundancy may exist in *S. maltophilia*. *P. aeruginosa* was the first organism known to encode two functional heme oxygenases, PigA and BphO, each capable of oxidizing heme to a different biliverdin isomer. PigA oxidizes heme into a mixture of β- and δ-biliverdin (Caignan et al., 2002), whereas BphO favors the formation of α-biliverdin (Wegele et al., 2004), similarly to the heme oxygenases of *Neisseria meningitidis* (HemO) (Zhu et al., 2000). The protein identity between PigA and BphO is as low as 24%. Of the two heme oxygenases, PigA plays a major role in iron supply (Ratliff et al., 2001). Based on this rationale, we attempted to survey the putative heme oxygenase in *S. maltophilia* using the known heme oxygenases of other bacteria, listed in **Figure 4**, as queries; however, no positive results were obtained. Thus, the exact mechanism responsible for the release of iron from hemin in *S. maltophilia* requires further investigation.

Iron complex uptake via the outer membrane TBDT is an energy-dependent process that relies on the inner membrane TonB-ExbB-ExbD complex. TonB-ExbB-ExbD uses a proton gradient across the inner membrane as an energy source to transport the iron complex from the extracellular environment to the periplasm (Torres and Payne, 1997). TonB is a well-structured protein with an N-terminal transmembrane helix anchored to the inner membrane, a proline-rich periplasmic domain, and a C-terminal domain that interacts with the TonB box of TBDTs (Kohler et al., 2010). The number of *tonB* genes varies significantly among different species, being as low as one in *E. coli* and up to 15 homologs in *Bacteroides thetaiotaomicron* (Chu et al., 2007). High sequence diversity has been observed across the characterized TonB proteins, even within the same species, suggesting that each TonB protein functions differently. Nevertheless, functional redundancy has also been reported in TonBs (Liao et al., 2015). Thus, multiple TonB proteins in an organism can function in redundant or unique manners. A genome-wide survey of the *S. maltophilia* K279a genome revealed that *S. maltophilia* harbors 10 annotated *tonB* genes (Crossman et al., 2008). Among the 10 *tonB* homologs, only *tonB1* (Smlt0009) and *tonB2* (Smlt3892) were linked to and in the same transcriptional orientation as *exbB* and *exbD* (**Supplementary Figure S2**). The only previous study of *S. maltophilia* TonBs showed linkage between TonB1 and β-lactam susceptibility (Calvopiña et al., 2020). In this study, we investigated the involvement of TonB1 and TonB2 in hemin acquisition and found that TonB1 predominantly contributed to hemin acquisition. Although *tonB2* and *hemU* are organized into an operon, our results do not support the involvement of TonB2 in hemin acquisition.

**Figure 5:**
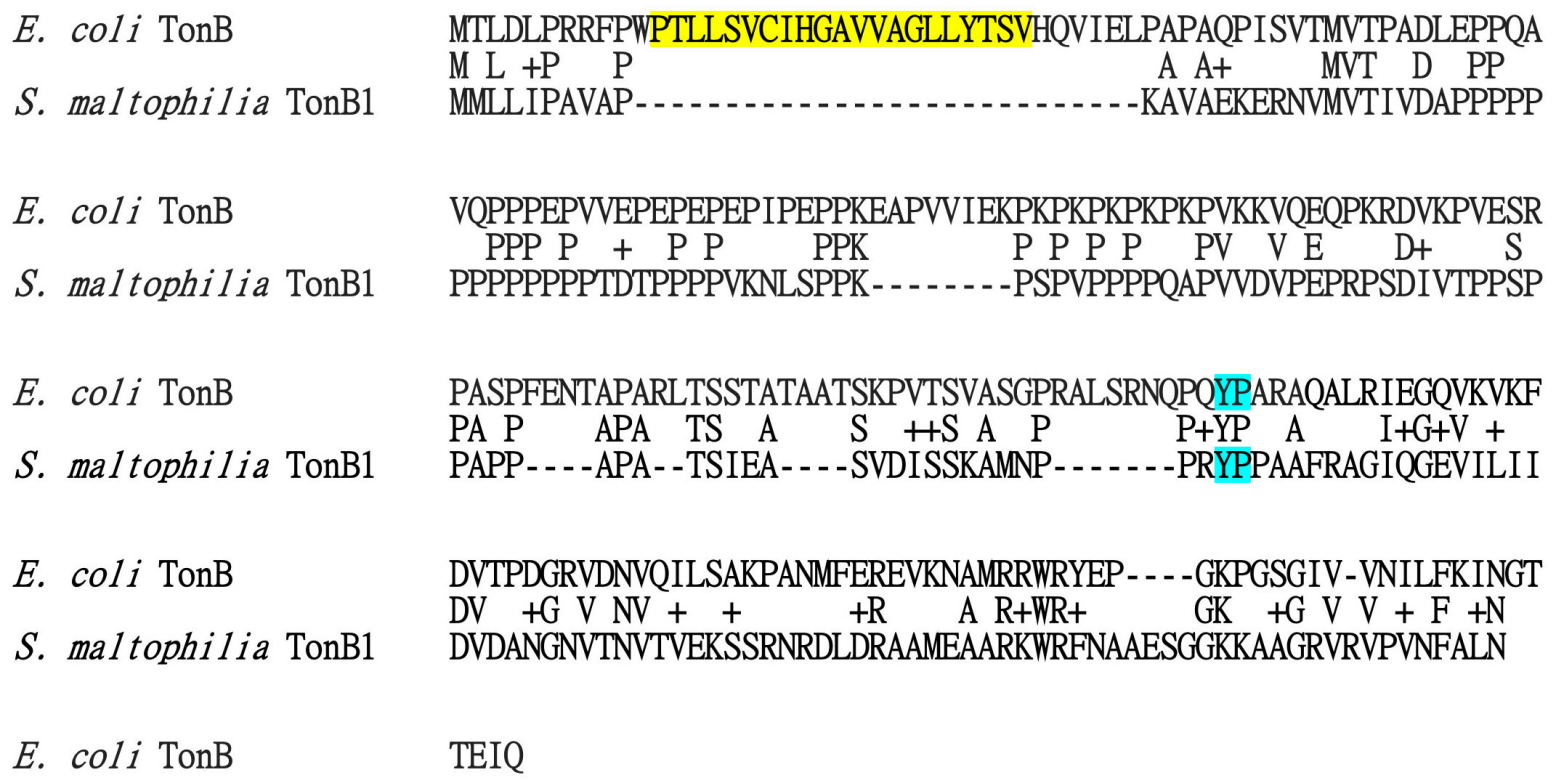
Protein sequence alignment of *Esherichia coli* TonB and *S. maltophilia* TonB1. Protein sequence alignment was carried out using Needleman-Wunsch global alignment using sequences in the National Center for Biotechnology Information database. The putative hydrophobic transmembrane region of *E. coli* TonB, amino acid residues 12–32, is marked in yellow. The most conserved YP motif among the TonB proteins is marked in blue. The protein accession numbers for *E. coli* TonB and *S. maltophilia* TonB1 are EOW16097 and CAQ43627, respectively.

Of note, the 185-aa TonB1 protein has a relatively short N-terminus. Protein alignment between *E. coli* TonB and *S. maltophilia* TonB1 revealed that the putative hydrophobic transmembrane region of *E. coli* TonB (amino acid residues 12–32) (Jaskula et al., 1994), is absent in *S. maltotphilia* TonB1 (**Figure 5**). The N-terminus 1–39 residues of *E. coli* TonB are essential for its ability to transduce energy (Jaskula et al., 1994); however, this feature has not been observed in *S. maltophilia* TonB1. Thus, the N-terminal membrane anchor of TonB may not be the only mechanism by which TonB associates with the cytoplasmic membrane. In contrast, the YP motif is the most conserved feature among TonB proteins (Chu et al., 2007) and is conserved in *E. coli* TonB and *S. maltophilia* TonB1 (**Figure 5**).

Distinct from the known hemin permeases reported in other bacteria, such as HemU of *Y. enterocolitica*, PhuU of *P. aeruginosa*, and HmuUs of *B. japonicum*, *E. meliloti*, and *B. multivorans* (Shih et al., 2022), HemU of *S. maltophilia* owns some peculiar features. (i) Compared to the other hemin permeases of 326-372 amino acids, HemU of *S. maltophilia* is relative shorter (155 amino acids) and displays no significant protein similarity with the known hemin permease. *S. maltophilia* HemU can be a novel type of hemin permease. (ii) The role of HemU in hemin utilization is HemP dependent. In a *hemP* mutant, there is an unidentified HemU-independent iron utilization mechanism.

**Figure 6:**
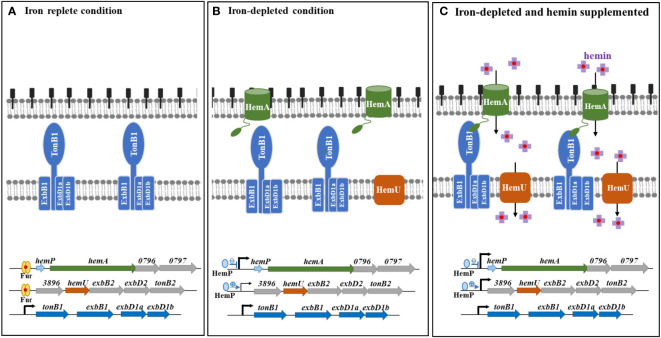
The proposed model for hemin acquisition by *S. maltophilia*. **(A)** In iron-replete conditions, *hemP-hemA-Smlt0796-Smlt0797* and *Smlt3896-hemU-exbB2-exbD2-tonB2* operons are repressed by Fur. Nevertheless, *tonB1-exbB1-exbD1a-exbD1b* operon is constitutively expressed. **(B)** In iron-depleted conditions, *hemP-hemA-Smlt0796-Smlt0797* and *Smlt3896-hemU-exbB2-exbD2-tonB2* operons are derepressed due to the loss-of-function of Fur. Meanwhile, HemP has negative and positive roles in the regulation of *hemP-hemA-Smlt0796-Smlt0797* and *Smlt3896-hemU-exbB2-exbD2-tonB2* operons, respectively. **(C)** In iron-depleted and hemin-supplemented condition, *Smlt3896-hemU-exbB2-exbD2-tonB2* operon is further upregulated. Hemin is taken up via the outer membrane HemA receptor and TonB1-ExbB1-ExbD1a-ExbD1b provides the energy. Hemin is then transported into the cytoplasm via the inner membrane transporter HemU.

The hemin acquisition and regulatory mechanisms in *S. maltophilia* are shown in **Figure 6**. Under iron-depleted conditions, hemin is loaded onto the outer membrane of TBDT HemA, followed by subsequent delivery into the periplasmic space in an energy-dependent manner. This energy for this process is provided by the TonB1-ExbB1-ExbD1a-ExbD1b complex. Hemin is then transported into the cytoplasm via the inner membrane transporter HemU. The genes involved in hemin acquisition are in three operons, *hemP-hemA-Smlt0796-Smlt0797*, *Smlt3896-hemU-exbB2-exbD2-tonB2*, and *tonB1-exbB1-exbD1a-exbD1b*. There is precedence for the coordination of the expression of these three operons. The *tonB1-exbB1-exbD1a-exbD1b* operon was constitutively expressed regardless of iron levels (**Figures 6A–C**), indicating that this operon may be involved in other physiological functions in addition to hemin acquisition. The involvement of TonB proteins in distinct functions other than iron acquisition has been reported, such as the involvement of *P. aeruginosa* TonB3 in twitching motility and type IV pili assembly (Huang et al., 2004), *S. maltophilia* TonB1 in β-lactam susceptibility (Calvopiña et al., 2020), and *Anabaena* sp. TonB2 in outer membrane integrity (Schätzle et al., 2021). The *hemP-hemA-Smlt0796-Smlt0797* operon is repressed by Fur in iron-replete conditions (**Figure 6A**). This operon was highly upregulated in response to iron-depletion stress, and HemP played a negative role in this regulatory circuit (**Figure 6B**). However, hemin did not significantly affect the expression of the *hemP-hemA-Smlt0796-Smlt0797* operon (**Figure 6C**). The expression of the *Smlt3896-hemU-exbB2-exbD2-tonB2* operon was regulated in a stepwise manner by Fur and HemP. In iron-rich situations, the *Smlt3896-hemU-exbB2-exbD2-tonB2* operon was repressed by Fur (**Figure 6A**). In response to iron-depleted stress, the *Smlt3896-hemU-exbB2-exbD2-tonB2* operon displays a moderate level of upregulation due to the loss-of-function of Fur and can be further fully expressed when hemin is present (**Figures 6B, C**). Compared with other known hemin acquisition systems (Shih et al., 2022), the periplasmic-binding proteins and HemO-like iron-releasing enzymes in *S. maltophilia* require further elucidation.

## Data availability statement

The original contributions presented in the study are included in the article/**Supplementary Materials**, further inquiries can be directed to the corresponding author.

## Author contributions

C-HL: Conceptualization, Funding acquisition, Investigation, Project administration, Writing – original draft, Writing – review & editing. H-FL: Data curation, Formal analysis, Methodology, Validation, Writing – review & editing. C-WY: Data curation, Formal analysis, Methodology, Writing – review & editing. T-YY: Data curation, Formal analysis, Methodology, Writing – review & editing. Y-TL: Data curation, Formal analysis, Methodology, Writing – review & editing. T-CY: Conceptualization, Data curation, Funding acquisition, Project administration, Supervision, Validation, Writing – review & editing.
