# Supplementary material for: HemU and TonB1 contribute to hemin acquisition in Stenotrophomonas maltophilia
Source: Front Cell Infect Microbiol. 2024 Mar 26;14:1380976. doi: 10.3389/fcimb.2024.1380976 (PMC11002078; doi:10.3389/fcimb.2024.1380976)
Supplement: Supplementary file 1 [file DataSheet_1.pdf]

(A)

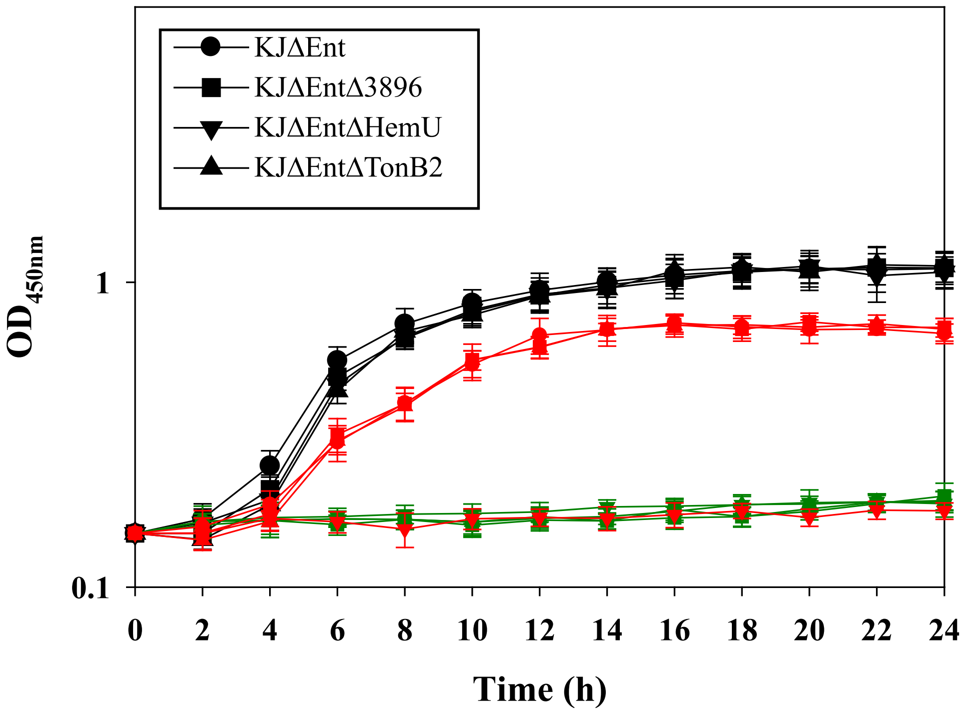
KJΔEnt
KJΔEntΔ3896
KJΔEntΔHemU
KJΔEntΔTonB2
OD450nm
1
0.1
0
2
4
6
8
10
12
14
16
18
20
22
24
Time (h)

(B)

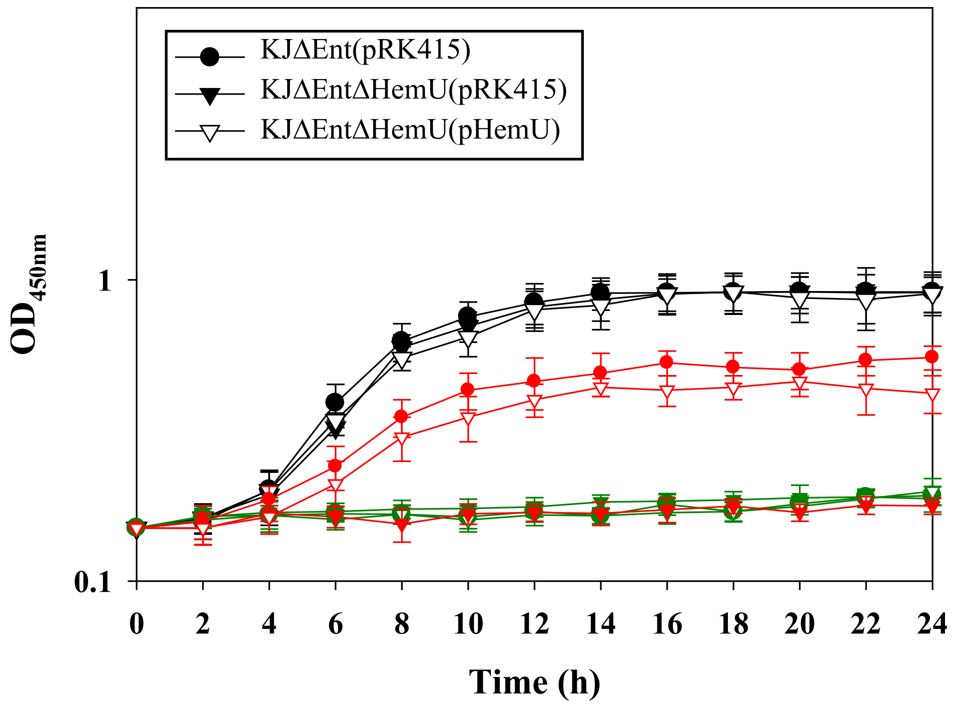
KJΔEnt(pRK415)
KJΔEntΔHemU(pRK415)
KJΔEntΔHemU(pHemU)
OD450nm
1
0.1
0
2
4
6
8
10
12
14
16
18
20
22
24
Time (h)

(C)

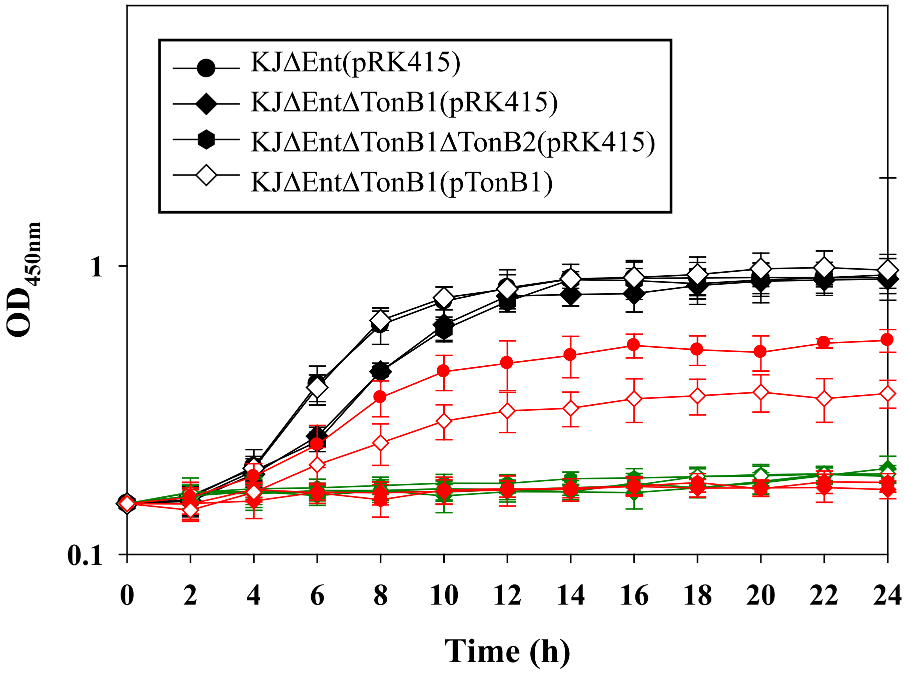
KJΔEnt(pRK415)
KJΔEntΔTonB1(pRK415)
KJΔEntΔTonB1ΔTonB2(pRK415)
KJΔEntΔTonB1(pTonB1)
OD450nm
1
0.1
0
2
4
6
8
10
12
14
16
18
20
22
24
Time (h)

(D)

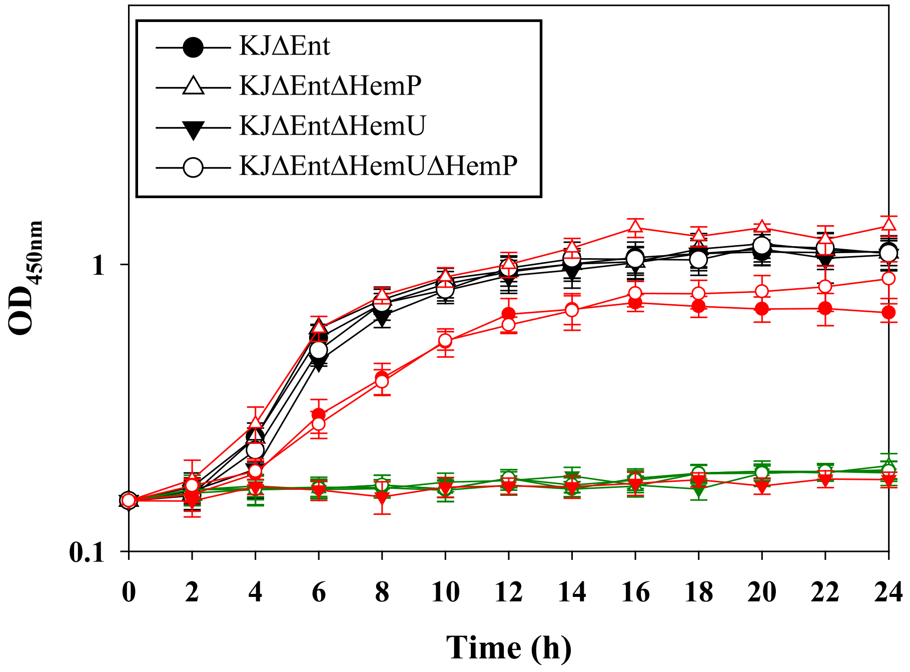
KJΔEnt
KJΔEntΔHemP
KJΔEntΔHemU
KJΔEntΔHemUΔHemP
OD450nm
1
0.1
0
2
4
6
8
10
12
14
16
18
20
22
24
Time (h)

Fig. S1. Growth curve of parental strain KJΔEnt, its derived mutants, and complementation strains under iron-replete (LA), iron-depleted (LA + DIP), and iron-depleted with hemin as sole iron source (LA + DIP + hemin). An overnight bacterial culture was inoculated to the LB broth as indicated at an initial OD_{450} nm of 0.15. Bacterial growth was monitored by recording the OD_{450} nm for 24 h at intervals of 2 h. For complementation test, the complemented gene was cloned in plasmid pRK415. Black, green, and red symbols and lines indicate LB, LB plus DIP, and LB plus DIP and hemin, respectively. DIP, 50 μg/mL; hemin, 150 μM. (A) Role of *smlt3896-hemU-exbB2-exbD2-tonB2* operon in hemin utilization. (B) Role of *hemU* in hemin utilization. (C) Role of *tonB1* and *tonB2* in hemin utilization. (D) Role of *hemP* and *hemU* in hemin utilization.
