# Supplementary material for: HemU and TonB1 contribute to hemin acquisition in Stenotrophomonas maltophilia
Source: Front Cell Infect Microbiol. 2024 Mar 26;14:1380976. doi: 10.3389/fcimb.2024.1380976 (PMC11002078; doi:10.3389/fcimb.2024.1380976)
Supplement: Supplementary file 2 [file DataSheet_2.pdf]

(A)

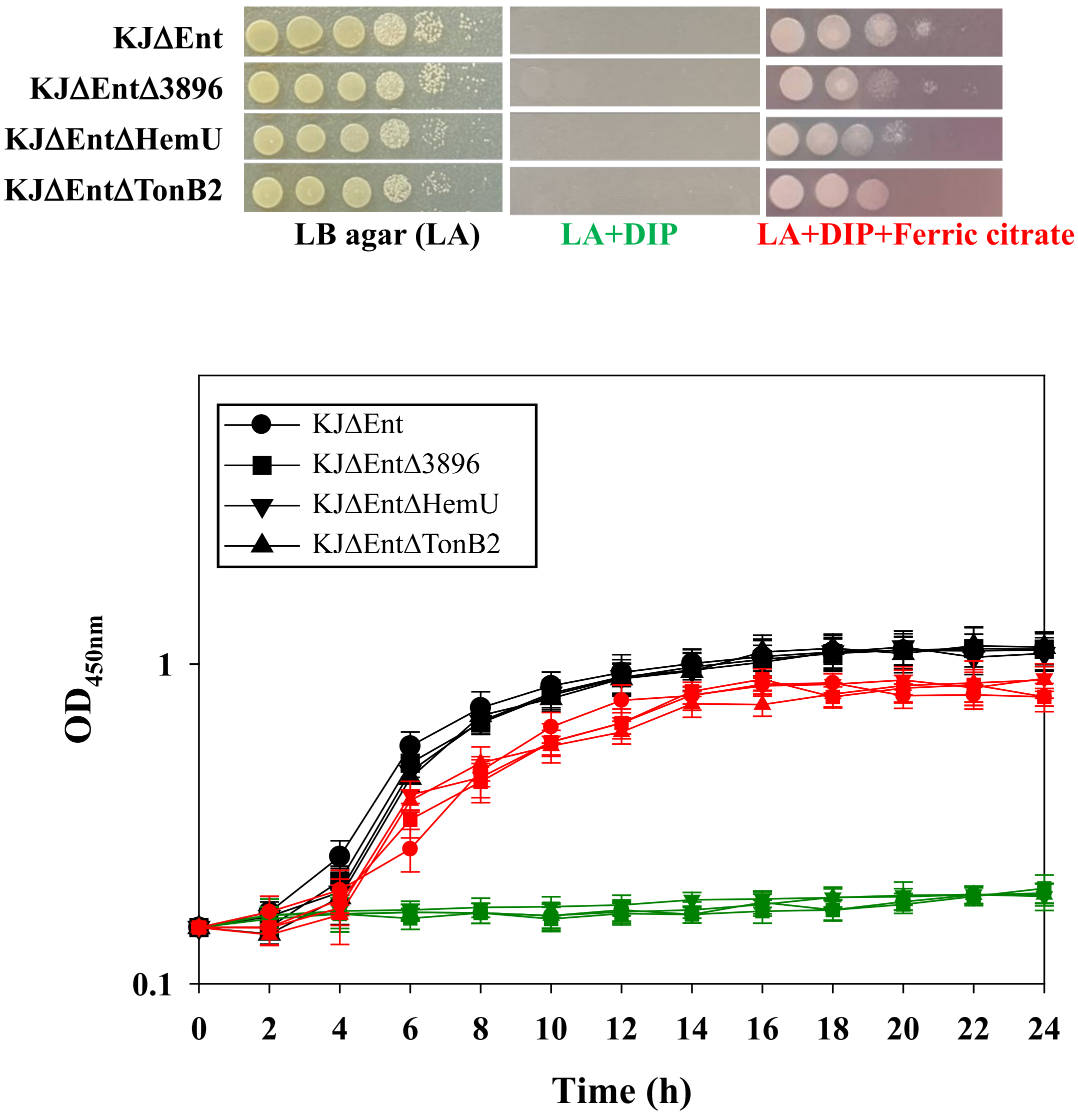
KJΔEnt
KJΔEntΔ3896
KJΔEntΔHemU
KJΔEntΔTonB2
LB agar (LA)
LA+DIP
LA+DIP+Ferric citrate
KJΔEnt
KJΔEntΔ3896
KJΔEntΔHemU
KJΔEntΔTonB2
OD_{450nm}
1
0.1
0
2
4
6
8
10
12
14
16
18
20
22
24
Time (h)

(B)

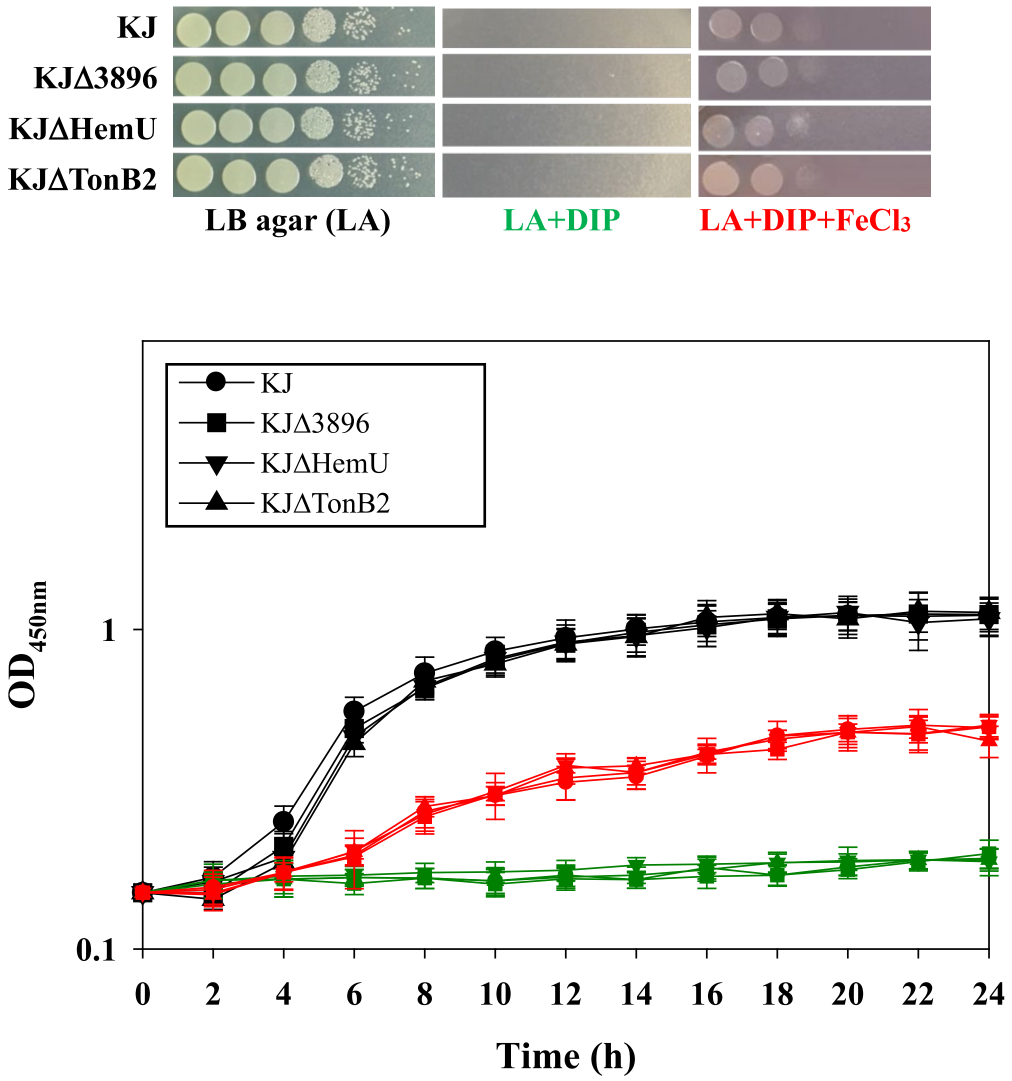

Fig. S2. Role of *smlt3896-hemU-exbB2-exbD2-tonB2* operon in ferric citrate and ferri-stenobactin acquisition. Bacterial cells of 2×10^5 CFU/μL were 10-fold serial diluted and 5 μL bacterial aliquot was spotted onto LB agar as indicated. After a 24-h incubation at 37°C, the bacterial viability was imaged. The experiment was performed in triplicate, and one was selected as a representative. For bacterial growth in liquid medium, an overnight bacterial culture was inoculated to LB broth as indicated at an initial OD_{450} nm of 0.15. Bacterial growth was monitored by recording the OD_{450nm} for 24 h at intervals of 2 h. Black, green, and red symbols and lines indicate LB, LB plus DIP, and LB plus DIP and ferric citrate (or $FeCl_3$), respectively. DIP, 50 μg/mL; ferric citrate, 110 μM; $FeCl_3$, 35 μM. (A) Role of *smlt3896-hemU-exbB2-exbD2-tonB2* operon in ferric citrate utilization. (B) Role of *smlt3896-hemU-exbB2-exbD2-tonB2* operon in ferri-stenobactin utilization.
