# Supplementary material for: HemU and TonB1 contribute to hemin acquisition in Stenotrophomonas maltophilia
Source: Front Cell Infect Microbiol. 2024 Mar 26;14:1380976. doi: 10.3389/fcimb.2024.1380976 (PMC11002078; doi:10.3389/fcimb.2024.1380976)
Supplement: Supplementary file 3 [file DataSheet_3.pdf]

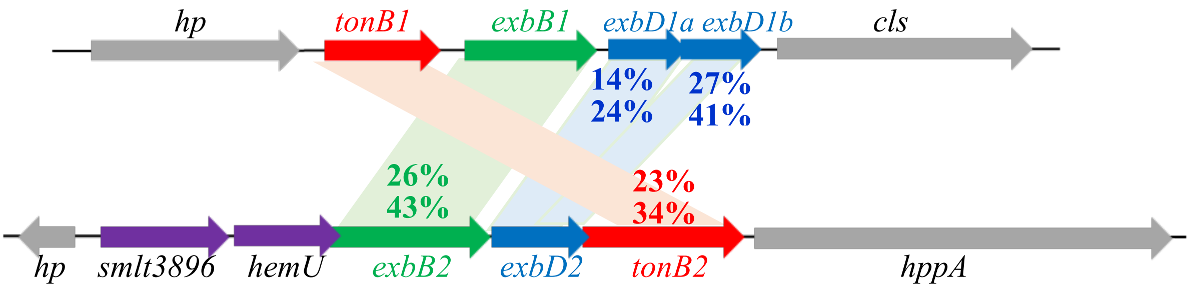

Fig. S3. The genetic organizations of *tonB1-exbB1-exbD1a-exbD1b* and *exbB2-exbD2-tonB2* clusters of *S. maltophilia*. The orientation of the gene is indicated by an arrow. Genes encoding the *exbB*, *exbD*, and *tonB* are marked in green, blue, and red, respectively. The numbers labelled in the shaded areas indicate the protein identities and similarities between the two proteins compared.
