# Supplementary material for: HemU and TonB1 contribute to hemin acquisition in Stenotrophomonas maltophilia
Source: Front Cell Infect Microbiol. 2024 Mar 26;14:1380976. doi: 10.3389/fcimb.2024.1380976 (PMC11002078; doi:10.3389/fcimb.2024.1380976)
Supplement: Supplementary file 4 [file DataSheet_4.pdf]

Table S1 Bacterial strains and plasmids used in this study

Strain or plasmid	Genotype or properties	Reference
S. maltophilia		
KJ	A clinical *S. maltophilia* isolate	Hu et al. 2008
KJΔFur	*S. maltophilia* KJ mutant of *fur* gene; *Δfur*	Shih et al. 2022
KJΔHemP	*S. maltophilia* KJ mutant of *hemP* gene; *ΔhemP*	Shih et al. 2022
KJΔEnt	*S. maltophilia* KJ mutant of *entF* and *entA* genes; *ΔentF, ΔentA*	Liao et al. 2020
KJΔEntΔHemA	*S. maltophilia* KJΔEnt mutant of *hemA* gene; *ΔentF, ΔentA, ΔhemA*	Shih et al. 2022
KJΔEntΔ3896	*S. maltophilia* KJΔEnt mutant of *smlt3896* gene; *ΔentF, ΔentA, Δsmlt3896*	This study
KJΔEntΔHemU	*S. maltophilia* KJΔEnt mutant of *hemU* gene; *ΔentF, ΔentA, ΔhemU*	This study
KJΔEntΔTonB2	*S. maltophilia* KJΔEnt mutant of *tonB2* gene; *ΔentF, ΔentA, ΔtonB2*	This study
KJΔEntΔTonB1	*S. maltophilia* KJΔEnt mutant of *tonB1* gene; *ΔentF, ΔentA, ΔtonB1*	This study
KJΔEntΔTonB1ΔTonB2	*S. maltophilia* KJΔEnt mutant of *tonB1* and *tonB2* gene; *ΔentF, ΔentA, ΔtonB1, ΔtonB2*	This study
E. coli		
DH5α	F- φ80d/*acZΔM15* Δ(*lacZYA-argF*)*U169 deoR recA1 endA1 hsdR17* (r_k^- m_k^+) *phoA supE44λ thi-1 gyrA96 relA1*	Invitrogen
S17-1	λ*pir*$^+$ mating strain	Simon et al. 1986

**Plasmids**		
pEX18Tc	*sacB oriT*, Tc^r	Hoang et al. 1998
pRK415	Mobilizable broad-host-range plasmid cloning vector, RK2 origin; Tc^r	Keen et al. 1988
pΔ3896	pEX18Tc with an internal-deleted *smlt3896* gene; Tc^r	This study
pΔHemU	pEX18Tc with an internal-deleted *hemU* gene; Tc^r	This study
pΔTonB1	pEX18Tc with an internal-deleted *tonB1* gene; Tc^r	This study
pΔTonB2	pEX18Tc with an internal-deleted *tonB2* gene; Tc^r	This study
pHemU	pRK415 with an intact *hemU* gene; Tc^r	This study
pTonB1	pRK415 with an intact *tonB1* gene; Tc^r	This study

Reference

Hoang, T. T., Karkhoff-Schweizer, R. R., Kutchma, A. J., and Schweizer, H. P. (1998). A broad-host-range Flp-FRT recombination system for site-specific excision of chromosomally-located DNA sequences: application for isolation of unmarked *Pseudomonas aeruginosa* mutants. *Gene.* 212, 77-86.

Hu, R. M., Huang, K. J., Wu, L. T., Hsiao, Y. J., and Yang, T. C. (2008). Induction of L1 and L2 β-lactamases of *Stenotrophomonas maltophilia*. *Antimicrob Agents Chemother.* 52, 1198-1200.

Keen, N. T., Tamaki, S., Kobayashi, D., and Trollinger, D. (1988). Improved broad-host-range plasmids for DNA cloning in gram-negative bacteria. *Gene.*70, 191-197.

Liao, C. H., Chen, W. C., Li, L. H., Lin, Y. T., Pan, S. Y., and Yang, T. C. (2020). AmpR of *Stenotrophomonas maltophilia* is involved in stenobactin synthesis and enhanced β-lactam resistance in an iron-depleted condition. *J. Antimicrob. Chemother.* 75, 3544–3551.

Shih, Y. L., Wu, C. M., Lu, H. F., Li, L. H., Lin, Y. T., and Yang, T. C. (2022). Involvement of the *hemP-hemA-smlt0796-smlt0797* operon in hemin acquisition by *Stenotrophomonas maltophilia*. *Microbiol Spectr.* 10, e0032122.

Simon, R., O'Connell, M., Labes, M., and Puhler, A. (1986). Plasmid vector for the genetic analysis and manipulation of *Rhizobia* and other Gram-negative bacteria. *Methods Enzymol.* 118, 640-659.
