# Supplementary material for: HemU and TonB1 contribute to hemin acquisition in Stenotrophomonas maltophilia
Source: Front Cell Infect Microbiol. 2024 Mar 26;14:1380976. doi: 10.3389/fcimb.2024.1380976 (PMC11002078; doi:10.3389/fcimb.2024.1380976)
Supplement: Supplementary file 5 [file DataSheet_5.pdf]

Table S2 Primers used in this study

primer	Sequence (5'→3')	Purpose	Reference
3896N-F<br>3896N-R<br>HemU-F<br>HemU-R	TCGAAGCTTTTACCGCATCTTC<br>CGGGCATGCTGCGCTTGTCCAG<br>GTGGCATGCGCCTGATGCAC<br>AGCGAGCTCATCACGGCCTTCACC	pΔ3896 construction	This study
3896-F<br>3896-R<br>3894-F<br>3894-R	GCCAAGCTTCAGGAACAGGTTGGTC<br>AGCTCTAGACAGAGCCAGCGCATC<br>TGTTCTAGATGGAAACCGAGACGAT<br>CGTGAGCTCCTTCCGGTGCGTCGT	pΔHemU construction	This study
TonB2N-F<br>TonB2N-R<br>TonB2C-F<br>TonB2C-R	GCGAAGCTTTATGTGACCGTGCAG<br>CTGCTGCAGTGGACCGGTGGCG<br>CACCTGCAGAAGCATCGCCGGC<br>CCTGGGAGAATTCCAAGAAGTACA	pΔTonB2 construction	This study
TonB1N-F<br>TonB1N-R<br>TonB1C-N<br>TonB1C-R	CATCTAGAAGCCGGACTACCA<br>CTTGGTACCGGGTTGTTCGTA<br>AAGGTACCGGTAAGAAGACG<br>GGGAATTCCAGAACAGGCTGA	pΔTonB1 construction	This study
HemU-F<br>HemU-R	GTGGCATGCGCCTGATGCAC<br>AGCGAGCTCATCACGGCCTTCACC	pHemU construction	This study
TonB1-F<br>TonB1-R	CATCTAGAAGCCGGACTACCA<br>GGGAATTCCAGAACAGGCTGA	pTonB1 construction	This study
3896Q-F<br>3896Q-R	CGTCCGATCTGGAACTGC<br>AGCTTGGCAGCCATCTTGTA	operon verification	This study
HemUQ-F<br>HemUQ-R	ATGCGCTGGCTCTGGTTC<br>CCAGGATCAGGAAGATGGTG	operon verification<br>qRT-PCR	This study
ExbB2Q-F<br>ExbB2Q-R	CAGAGCGAAGAGGCAAGTCT<br>TATCCAGATCCTGCGACAGA	operon verification	This study
ExbB2-C	GATGAAACTGTTCATGATGCC	RT	This study
TonB2Q-F<br>TonB2Q-R	GAGGCAAGCTGGGAAGGTC<br>CAAAGCGCACGTAGACCAC	operon verification	This study
HemAQ93-F<br>HemAQ93-R	CCTGCTCAGCAAACTGGTCT<br>AGCACATTGGTATCGGTGGT	qRT-PCR	Shih et al., 2022
TonB1Q100-F<br>TonB1Q100-R	CGGTACGAACAACCCGATGA<br>TGAGGAGCATCATGAAGGCG	qRT-PCR	This study
16S rDNA-F	GACCTTGCGCGATTGAATG	qRT-PCR	Chen et al.

16S rDNA-R	CGGATCGTCGCCTTGGT		2011

Reference

Chen, C. H., Huang, C. C., Chung, T. C., Hu, R., M., Huang, Y. W., and Yang, T. C. (2011). Contribution of resistance-nodulation-division efflux pump operon *smeU1-V-W-U2-X* to multidrug resistance of *Stenotrophomonas maltophilia*. *Antimicrob Agents Chemother*. **55,** 5826-5833**.**

Shih, Y. L., Wu, C. M., Lu, H. F., Li, L. H., Lin, Y. T., and Yang, T. C. (2022). Involvement of the *hemP-hemA-smlt0796-smlt0797* operon in hemin acquisition by *Stenotrophomonas maltophilia*. *Microbiol Spectr*. 10, e0032122.
